# A Treatise on Diseases of the Nervous System

**Published:** 1871-08

**Authors:** 


### Book Notices.

A Treatise on Diseases of the Nervous System, by William A. Hammond, M.D., Professor of Diseases of the Mind and Nervous System, and of Clinical Medicine, in the Bellevue Hospital Medical College, etc. New York: D. Appleton & Co.; Chicago: S. C. Griggs & Co.

This is a handsome volume of 750 pages, in substantial cloth binding. The author has endeavored, as he states in the preface, "to present a Treatise on Diseases of the Nervous System, which, without being superficial, would be concise and explicit, and which, while making no claim to be exhaustive, would nevertheless be sufficiently complete for the instruction and guidance of those who might be disposed to seek information from its pages."

This volume, in connection with the fourth volume of Prof. Austin Flint, Jr.'s, Physiology of Man, which will be published during the coming season, is intended to constitute a complete work on the "Physiology and Pathology of the Nervous System." The author has views of his own on every disease considered, opinions founded to a great extent upon his own observation and experience. The name and reputation of Prof. Hammond is, of itself, a sufficient guarantee of the value and importance of these opinions. We shall endeavor to give a more extended review of the work in a subsequent number.

